# T239. SINGLE-SUBJECT PREDICTION OF FUNCTIONAL OUTCOMES IN CLINICAL HIGH RISK SUBJECTS USING CLINICAL DATA

**DOI:** 10.1093/schbul/sby016.515

**Published:** 2018-04-01

**Authors:** Marlene Rosen, Nathalie Kaiser, Theresa Haidl, Mauro Seves, Frauke Schultze-Lutter, Stefan Borgwardt, Paolo Brambilla, Eva Meisenzahl, Christos Pantelis, Stephan Ruhrmann, Raimo Salokangas, Rachel Upthegrove, Stephen Wood, Nikolaos Koutsouleris, The PRONIA Group

**Affiliations:** 1 University of Cologne; 2 Heinrich-Heine University Düsseldorf; 3 UPK, University of Basel; 4 University of Milan; 5 Melbourne Neuropsychiatry Centre, The University of Melbourne; 6 University of Turku; 7 University of Birmingham; 8 Orygen, the National Centre of Excellence in Youth Mental Health; 9 Ludwig Maximilian University

## Abstract

**Background:**

Psychotic disorders are associated with serious deterioration in functioning even before the first psychotic episode. Also on clinical high risk (CHR) states of developing a first psychotic episode, several studies reported a decreased global functioning. In a considerable proportion of CHR individuals, functional deterioration remains even after (transient) remission of symptomatic risk indicators. Furthermore, deficits in functioning cause immense costs for the health care system and are often more debilitating for individuals than other symptoms. However in the past, CHR research has mostly focused on clinical outcomes like transition and therefore, functioning in CHR patients is under-investigated. The current study aims at predicting functioning at a single subject level applying multi pattern recognition to clinical data for the first time.

**Methods:**

PRONIA (‘Personalized Prognostic Tools for Early Psychosis Management’) is a prospective collaboration project funded by the European Union under the 7th Framework Programme (grant agreement n° 602152). Considering a broad set of variables (sMRI, rsMRI, DTI, psychopathological, life event related and sociobiographic data, neurocognition, genomics and other blood derived parameters) as well as advanced statistical methods, PRONIA aims at developing an innovative multivariate prognostic tool enabling an individualized prediction of illness trajectories and outcome. Seven university centers in five European countries and in Australia (Munich, Basel, Birmingham, Cologne, Melbourne, Milan/Udine, Turku) participate in the evaluation of three clinical groups (subjects clinically at high risk of developing a psychosis [CHR], patients with a recent onset psychosis [ROP] and patients with a recent onset depression [ROD]) as well as healthy controls.

In the current study, we analysed data of 114 CHR patients. Functioning was measured by the ‘Global Functioning: Social and Role’ Scales (GF S/R). Features were derived from the large pool of clinical data that were assessed in PRONIA including questionnaires measuring CHR criteria as well as psychopathology, family history of psychotic disorders or treatment and various self-rating scales. Feature Elimination method of a strict Wrapper was used to identify most predictive variables from the multitude of clinical data included into the analysis.

**Results:**

Balanced Accuracy of predicting social functioning in CHR patients was acceptable (pooled cross-validation: BAC = 74.3%, Sens = 72.8%, Spec = 60.3%; leave-site-out cross-validation: BAC = 69.9%, Sens = 84.3%, Spec = 55.6%). In contrast, applying the strict wrapper model revealed worse prediction performance for role functioning. Which might indicate that predicting level of role functioning requires more information than social functioning. As expected, prior functioning levels were identified as main predictive factor but also distinct protective and risk factors were selected into the prediction models.

**Discussion:**

Identifying single predictive variables is in purpose of a much more efficient prognostic process. Moreover, understanding the mechanisms underlying functional decline and its illness related pattern might enable an improved definition of targets for intervention. Future research should aim at further maximisation of prediction accuracy and cross-centre generalisation capacity. In addition, other functioning outcomes as well as clinical outcomes need to be focused on.

